# Association between initial in-hospital heart rate and glycemic control in patients with acute ischemic stroke and diabetes mellitus

**DOI:** 10.1186/s12902-023-01325-2

**Published:** 2023-03-29

**Authors:** Ya-Wen Kuo, Jiann-Der Lee, Chuan-Pin Lee, Yen-Chu Huang, Meng Lee

**Affiliations:** 1 Department of Nursing, Chang Gung University of Science and Technology, Chiayi Campus, Chiayi, Taiwan; 2 Chiayi Chang Gung Memorial Hospital, Chiayi, Taiwan; 3 Department of Neurology, Chiayi Chang Gung Memorial Hospital, No.6, W. Sec., Jiapu Rd., Puzi City, Chiayi County 613, Chiayi, Taoyuan, Taiwan (R.O.C.); 4 College of Medicine, Chang Gung University, Taoyuan, Taiwan; 5 Health Information and Epidemiology Laboratory, Chiayi Chang Gung Memorial Hospital, Chiayi, Taiwan

**Keywords:** Acute ischemic stroke, Diabetes mellitus, Heart rate

## Abstract

**Background:**

A high resting heart rate (HR) has been associated with an increased risk of diabetes mellitus. This study explored the association between initial in-hospital HR and glycemic control in patients with acute ischemic stroke (AIS) and diabetes mellitus.

**Methods:**

We analyzed data from 4,715 patients with AIS and type 2 diabetes mellitus enrolled in the Chang Gung Research Database between January 2010 and September 2018. The study outcome was unfavorable glycemic control, defined as glycated hemoglobin (HbA1c) ≥ 7%. In statistical analyses, the mean initial in-hospital HR was used as both a continuous and categorical variable. Odds ratios (ORs) and 95% confidence intervals (CIs) were estimated using multivariable logistic regression analysis. The associations between the HR subgroups and HbA1c levels were analyzed using a generalized linear model.

**Results:**

Compared with the reference group (HR < 60 bpm), the adjusted ORs for unfavorable glycemic control were 1.093 (95% CI 0.786–1.519) for an HR of 60–69 bpm, 1.370 (95% CI 0.991–1.892) for an HR of 70–79 bpm, and 1.608 (95% CI 1.145–2.257) for an HR of ≥ 80 bpm. Even after adjusting for possible confounders, the HbA1c levels after admission and discharge among diabetic stroke patients increased significantly in the subgroups with higher HRs (*p* < 0.001).

**Conclusions:**

High initial in-hospital HR is associated with unfavorable glycemic control in patients with AIS and diabetes mellitus, particularly in those with an HR of ≥ 80 bpm, compared with those with an HR of < 60 bpm.

## Background

Diabetes mellitus is a chronic disease with long-term complications, and the development of hyperglycemia is common in individuals with diabetes mellitus after an acute stroke [1]. Although the relationship between the control of hyperglycemia and cardiovascular risk remains largely controversial [2], glycemic control remains crucial to managing diabetes mellitus. The American Diabetes Association recommends a target glycated hemoglobin (HbA1c) level of < 7% for most adult patients [3]. In stroke patients with diabetes, medical therapies and the goal of glycemic control should be individualized, but for most patients, an HbA1c level of < 7% is also recommended [4]. However, only approximately 50% of patients with diabetes mellitus reach their HbA1c target [5]. The reasons for poor glycemic control among patients with type 2 diabetes mellitus are complex and multivariable.

A high resting heart rate (HR) is associated with an increased risk of type 2 diabetes mellitus [6–9] and poor glycemic status [10]. Increased levels of HbA1c and insulin dose are associated with increased diastolic blood pressure and HR in children with type 1 diabetes mellitus [11]. Compared with conventional therapy, the intensive management of diabetes mellitus is associated with a lower resting HR in patients with type 1 diabetes mellitus [12]. However, the association between resting HR and glycemic control in patients with acute ischemic stroke (AIS) and type 2 diabetes mellitus is seldom discussed.

This study evaluated the relationship between mean initial in-hospital HR and glycemic control in patients with AIS and type 2 diabetes mellitus.

## Methods

### Participants and data collection

We conducted this retrospective cohort study by using data from the Chang Gung Research Database [13], the largest multi-institutional collection of electronic medical records in Taiwan. Patients with AIS were selected per the method of Lee et al. [14]. A total of 21,655 patients with AIS between January 2010 and September 2018 were admitted to one of the seven branch hospitals of the Chang Gung Healthcare System. Patients with type 2 diabetes mellitus were defined as patients who, according to their medical records, received a diagnosis of type 2 diabetes mellitus after discharge, were administered drugs or insulin to treat hyperglycemia after admission, or had laboratory results that indicated the presence of type 2 diabetes mellitus, according to the American Diabetes Association [15]. Among 21,655 patients with AIS, 8,680 patients had type 2 diabetes mellitus, and 4,715 patients were followed up regularly at our outpatient clinics with at least three measurements of HbA1c levels after discharge. Ultimately, the data of 4,715 patients were used for this analysis. The study was conducted in accordance with the Declaration of Helsinki, and local ethical approval was obtained.

Primary demographic and clinical characteristics were collected, including stroke severity as assessed using the claims-based stroke severity index (SSI). The SSI was then converted to the National Institutes of Health Stroke Scale (NIHSS) score by using the following equation: estimated NIHSS (eNIHSS) = 1.1722 × SSI − 0.7533 [16]. Stroke severity was categorized into mild (eNIHSS score < 5), moderate (eNIHSS score 5–14), and severe (eNIHSS score > 14) [17]. The estimated glomerular filtration rate (eGFR) was determined using the Modification of Diet in Renal Disease equation as follows: eGFR = 186 × (serum creatinine level)^−1.154^ × (age)^−0.203^ × 0.742 (if female) [18]. Height, body weight, systolic blood pressure, diastolic blood pressure, HR, lipid profiles, and creatinine, alanine aminotransferase, and HbA1c levels after admission were obtained from the records of the enrolled patients. The patient’s history of cancer prior to admission was obtained from the Taiwan Cancer Registry [19]. Mean systolic blood pressure, diastolic blood pressure, and HR were calculated from vital sign measurements taken during the patient’s first 3 days of hospitalization. The mean HbA1c levels after discharge were calculated from the first three recorded HbA1c values after discharge. The intervals between HbA1c measurements accorded with the latest guidelines by the American Diabetes Association and National Institute for Health and Care Excellence [20, 21].

### Study outcomes

The study outcome was the status of glycemic control. Favorable glycemic control was defined as mean HbA1c < 7%, and unfavorable glycemic control was defined as mean HbA1c ≥ 7%.

### Statistical analysis

Quantitative variables are summarized as mean (standard deviation) or median (interquartile range), depending on the distribution of the data, and categorical variables are presented as number (percentage). Differences in demographic features between the patients with favorable and unfavorable glycemic control were evaluated using the student’s *t-*test or Wilcoxon’s rank sum test for continuous data and the chi-square test for categorical data.

To further evaluate the effect of initial in-hospital HR on the levels of HbA1c and glycemic control, the patients were classified into four subgroups according to mean HR (< 60, 60–69, 70–79, and ≥ 80 beats per minute [bpm]). Multivariable logistic regression analysis was used to evaluate the relationship between HR and unfavorable glycemic control. In statistical analyses, the mean initial in-hospital heart rate was used as both a continuous and categorical variable. In addition to crude odds ratios (ORs), adjusted ORs and 95% confidence intervals (CIs) were calculated using the HR < 60 bpm subgroup as the reference for the analysis. Model 1 included HR, age, and sex, and model 2 included HR, age, sex, stroke severity, body mass index, hypertension, dyslipidemia, atrial fibrillation, coronary artery disease, congestive heart failure, history of cancer before admission, smoking status, levels of total cholesterol, triglyceride, eGFR, and alanine aminotransferase, systolic blood pressure, diastolic blood pressure, and the use of beta blockers and insulin.

The influence of the initial in-hospital HR on the levels of HbA1c was assessed using the generalized linear model. We examined the effect of initial in-hospital HR on HbA1c level by adjusting for possible confounders, namely age, sex, stroke severity, body mass index, hypertension, dyslipidemia, atrial fibrillation, coronary artery disease, congestive heart failure, history of cancer before admission, smoking status, levels of total cholesterol, triglyceride, eGFR, and alanine aminotransferase, systolic blood pressure, diastolic blood pressure, and the use of beta blockers and insulin. All analyses were performed using SPSS for Windows, version 22 (SPSS Inc., Chicago, IL, USA).

## Results

### Participant demographics

A total of 4,715 patients with AIS and type 2 diabetes mellitus were included in this study (median age, 66 years, interquartile range: 59–74 years; 61.50% men). The median systolic blood pressure and diastolic blood pressure were 154.00 (interquartile range: 141.42–167.55 mmHg) and 85.13 mmHg (interquartile range: 78.70–92.33 mmHg), respectively, and the median HR was 74.71 bpm (interquartile range: 68.25–80.87 bpm) (Table 1). The intervals between discharge and the first, second, and third measurements of HbA1c levels were 87 (interquartile range: 46–149 days), 208 (interquartile range: 138–328 days), and 341 days (interquartile range: 237–507 days), respectively. Overall, 54.08% of the patients had unfavorable glycemic control after AIS. The values of HbA1c after admission and mean HbA1c after discharge were 7.80% (interquartile range: 6.80–9.60%) and 7.10% (interquartile range: 6.47–8.00%), respectively (Table 2).

**Table 1:** Comparison of clinical characteristics between the groups according to glycemic control

Clinical Background	Total (N = 4715)	Favorable glycemic control HbA1c < 7% (N = 2165)	Unfavorable glycemic control HbA1c ≥ 7% (N = 2550)	*p* value
Age (years)				< 0.001
Median (Q1, Q3)	66.00 (59.00, 74.00)	67.00 (59.00, 75.00)	65.00 (58.00, 73.00)	
Male	2898 (61.50%)	1381 (63.80%)	1517 (59.50%)	0.003
Stroke severity				< 0.001
Mild (eNIHSS < 5)	3617 (76.70%)	1604 (74.10%)	2013 (78.90%)	
Moderate (eNIHSS 5–14)	911 (19.30%)	445 (20.60%)	466 (18.30%)	
Severe (eNIHSS > 14)	187 (4.00%)	116 (5.40%)	71 (2.80%)	
BMI (kg/m^2^)				0.903
Median (Q1, Q3)	25.40 (23.15, 28.03)	25.35 (23.24, 28.04)	25.46 (23.08, 27.99)	
Hypertension	3961 (84.00%)	1861 (86.00%)	2100 (82.40%)	0.001
Dyslipidemia	2548 (54.00%)	1148 (53.00%)	1400 (54.90%)	0.198
Atrial fibrillation	432 (9.20%)	236 (10.90%)	196 (7.70%)	< 0.001
Coronary artery disease	495 (10.50%)	218 (10.10%)	277 (10.90%)	0.376
Congestive heart failure	210 (4.50%)	90 (4.20%)	120 (4.70%)	0.363
History of cancer	243 (5.20%)	128 (5.90%)	115 (4.50%)	0.030
Current smoker	1384 (29.40%)	604 (27.90%)	780 (30.60%)	0.043
Total cholesterol (mmol/L)				< 0.001
Median (Q1, Q3)	4.56 (3.86, 5.31)	4.48 (3.78, 5.23)	4.64 (3.96, 5.39)	
Triglyceride (mmol/L)				< 0.001
Median (Q1, Q3)	1.55 (1.12, 2.21)	1.46 (1.07, 2.08)	1.62 (1.18, 2.31)	
eGFR (mL/min/1.73 m^2^)				0.072
Median (Q1, Q3)	75.58 (53.14, 96.42)	74.68 (53.40, 94.70)	76.32 (52.92, 97.62)	
ALT (U/L)				0.660
Median (Q1, Q3)	21.00 (16.00, 31.00)	22.00 (16.00, 31.00)	21.00 (16.00, 30.00)	
SBP (mmHg)				0.689
Median (Q1, Q3)	154.00 (141.42, 167.55)	153.75 (141.14, 168.58)	154.07 (141.75, 166.77)	
DBP (mmHg)				0.161
Median (Q1, Q3)	85.13 (78.70, 92.33)	85.36 (78.74, 92.85)	85.00 (78.68, 92.00)	
Heart rate (bpm)				0.001
Median (Q1, Q3)	74.71 (68.25, 80.87)	74.25 (67.42, 80.31)	75.12 (69.00, 81.50)	
Beta blocker user	557 (11.80%)	282 (13.00%)	275 (10.80%)	0.018
Insulin user	345 (7.30%)	73 (3.40%)	272 (10.40%)	< 0.001

Quantitative variables are summarized as mean (standard deviation) or median (interquartile range), depending on the distribution of the data, and categorical variables are presented as number (percentage). Abbreviations: HbA1c, glycated hemoglobin; Q, quartile; eNIHSS, estimated National Institute of Health Stroke Scale; BMI, body mass index; eGFR, estimated glomerular filtration rate; ALT, alanine aminotransferase; SBP, systolic blood pressure; DBP, diastolic blood pressure; bpm, beats per minute

**Table 2:** HbA1c after admission and mean HbA1c and SD of HbA1c after discharge, according to mean heart rate subgroups

	Mean heart rate					*p* for trend
	Total (N = 4715)	< 60 bpm (N = 276)	60–69 bpm (N = 1167)	70–79 bpm (N = 1955)	≥ 80 bpm (N = 1317)	
HbA1c (%) after admission						< 0.001
Median (Q1, Q3)	7.80 (6.80, 9.60)	7.30 (6.50, 8.70)	7.60 (6.63, 9.10)	7.90 (6.90, 9.50)	8.10 (6.90, 10.10)	
Mean HbA1c (%) after discharge						< 0.001
Median (Q1, Q3)	7.10 (6.47, 8.00)	6.85 (6.33, 7.47)	7.00 (6.43, 7.90)	7.13 (6.50, 8.10)	7.20 (6.47, 8.17)	
SD of HbA1c (%) after discharge						< 0.001
Median (Q1, Q3)	0.37 (0.21, 0.69)	0.29 (0.17, 0.56)	0.36 (0.19, 0.63)	0.39 (0.21, 0.73)	0.40 (0.22, 0.74)	

Abbreviations: HbA1c, glycated hemoglobin; SD, standard deviation; bpm, beats per minute; Q, quartile

### Clinical factors associated with glycemic control

Univariate analysis of the data revealed that several clinical factors were significantly associated with glycemic control. These included age, sex, stroke severity, smoking status, HR, use of beta blockers and insulin after discharge, levels of total cholesterol and triglyceride, and history of hypertension, atrial fibrillation, and cancer before admission (Table 1). The levels of HbA1c after admission and mean HbA1c after discharge both increased progressively across the HR subgroups. The standard deviation values of the mean HbA1c levels after discharge also increased progressively across the HR subgroups (Table 2, *p* for trend < 0.001).

### Independent predictors of glycemic control

The results of the multivariable logistic regression analysis for unfavorable glycemic control are presented in Table 3. Age, sex, HR, severe stroke, smoking status, levels of total cholesterol, diastolic blood pressure, and the use of insulin after discharge were independently associated with glycemic control (Table 3).

**Table 3:** Multivariable logistic regression analysis with heart rate as a continuous variable for unfavorable glycemic control

Variables	Unfavorable glycemic control		
	OR	95% CI	*p* value
Heart rate	1.016	1.008–1.024	< 0.001
Age	0.984	0.976–0.992	< 0.001
Male (ref: female)	0.759	0.636–0.904	0.002
Stroke severity			
Mild (eNIHSS < 5)	reference		
Moderate (eNIHSS 5–14)	0.860	0.714–1.037	0.114
Severe (eNIHSS > 14)	0.407	0.276–0.598	< 0.001
Body mass index	0.995	0.976–1.015	0.626
HTN (ref: without HTN)	0.857	0.693–1.061	0.156
Dyslipidemia (ref: without dyslipidemia)	0.942	0.807–1.099	0.448
AF (ref: without AF)	0.916	0.705–1.190	0.511
CAD (ref: without CAD)	1.279	0.986–1.660	0.063
CHF (ref: without CHF)	1.286	0.893–1.853	0.177
History of cancer (ref: without history of cancer)	0.736	0.525–1.031	0.075
Smoker (ref: non-smoker)	1.200	1.001–1.439	0.048
Total cholesterol	1.081	1.005–1.162	0.035
Triglyceride	1.056	0.990–1.127	0.099
Estimated glomerular filtration rate	1.001	0.999–1.004	0.243
Alanine aminotransferase	0.998	0.994–1.001	0.123
Systolic blood pressure	1.005	0.999–1.011	0.096
Diastolic blood pressure	0.980	0.969–0.991	0.001
Beta blockers user (ref: non-user)	0.806	0.635–1.024	0.077
Insulin user (ref: non-user)	3.281	2.260–4.763	< 0.001

Abbreviations: OR, odds ratio; CI, confidence interval; ref, reference; eNIHSS, estimated National Institute of Health Stroke Scale; HTN, hypertension; AF, atrial fibrillation; CAD, coronary artery disease; CHF, congestive heart failure

Because a significant relationship was detected between the mean initial in-hospital HR and glycemic control, the associations between the mean initial in-hospital HR subgroups and HbA1c levels after admission as well as mean HbA1c levels after discharge were analyzed using a generalized linear model. Even after the possible confounders were adjusted for, the HbA1c levels after admission and discharge among stroke patients with diabetes increased significantly in the subgroups with higher HRs (Table 4, *p* < 0.001). These results suggested that a lower mean initial in-hospital HR was associated with better glycemic status.

**Table 4:** Glycemic status among patients with diabetes mellitus who have had a stroke, according to heart rate subgroups

	Mean heart rate				
	< 60 bpm	60–69 bpm	70–79 bpm	≥ 80 bpm	*p* value
HbA1c (%) after admission	7.72 ± 0.15^a^	8.10 ± 0.08^b^	8.46 ± 0.06^c^	8.78 ± 0.07^d^	< 0.001
Mean HbA1c (%) after discharge	7.06 ± 0.09^a^	7.26 ± 0.05^b^	7.40 ± 0.04^c^	7.49 ± 0.04^c^	< 0.001

Data are presented as mean ± standard error of the mean

^a, b, c, d^ Values in the same row that do not share the same superscript letter differ significantly

The model was adjusted for age, sex, stroke severity, body mass index, hypertension, dyslipidemia, atrial fibrillation, coronary artery disease, congestive heart failure, history of cancer before admission, smoking status, levels of total cholesterol, triglyceride, estimated glomerular filtration rate, and alanine aminotransferase, systolic blood pressure, diastolic blood pressure, and the use of beta blockers and insulin. Abbreviations: bpm, beats per minute, HbA1c, glycated hemoglobin

### Association between mean HR subgroup and glycemic control

**Fig. 1:**
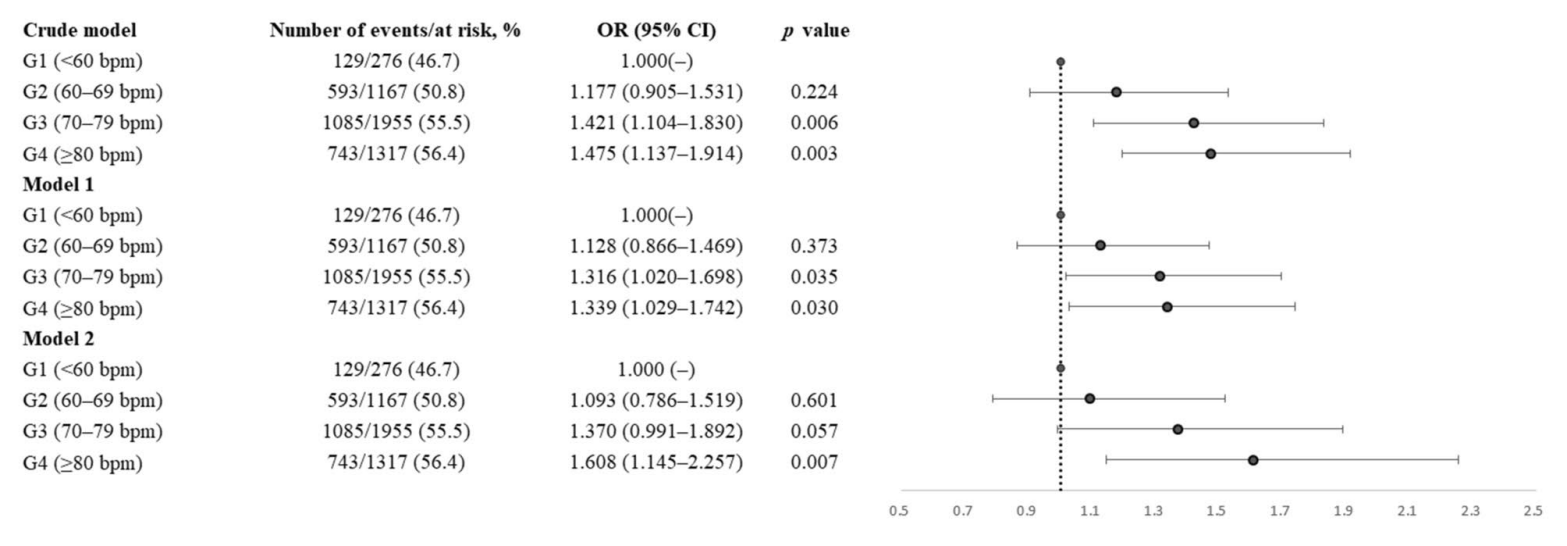
Forest plots of crude and adjusted odds ratios (95% CIs) for unfavorable glycemic control by mean initial in-hospital heart rate increments. The analyses were adjusted for age and sex in model 1 and for all of the variables in the fully adjusted model (model 2), including age, sex, stroke severity, body mass index, hypertension, dyslipidemia, atrial fibrillation, coronary artery disease, congestive heart failure, history of cancer before admission, smoking status, levels of total cholesterol, triglyceride, estimated glomerular filtration rate, and alanine aminotransferase, systolic blood pressure, diastolic blood pressure, and the use of beta blockers and insulin. Abbreviations: G, group; OR, odds ratio; CI, confidence interval; bpm, beats per minute

The crude and adjusted ORs for the HR subgroups are presented in Fig. 1. Relative to the reference group (HR < 60 bpm), the adjusted ORs for unfavorable glycemic control in model 2 were 1.093 (95% CI 0.786–1.519) for an HR of 60–69 bpm, 1.370 (95% CI 0.991–1.892) for an HR of 70–79 bpm, and 1.608 (95% CI 1.145–2.257) for an HR of ≥ 80 bpm (Fig. 1). A higher HR was associated with a lower probability of favorable glycemic control.

## Discussion

This study illustrated that an increased mean initial in-hospital HR was associated with unfavorable glycemic control in patients with AIS and type 2 diabetes mellitus, particularly in those with a mean initial in-hospital HR of ≥ 80 bpm, compared with those with an HR of < 60 bpm (Table 3; Fig. 1).

A higher resting HR in individuals without diabetes mellitus is associated with future unfavorable changes in insulin levels and insulin sensitivity [22, 23]. In the prospective RISC cohort study, HR predicted beta cell function and impaired glucose regulation after a 3-year follow-up in individuals without diabetes mellitus [24]. Proinsulin, acute insulin secretion, and insulin sensitivity were also associated with HR in individuals without diabetes mellitus [25]. According to National Health and Nutrition Examination Surveys, the mean HR increased with increasing HbA1c levels among individuals with diagnosed diabetes mellitus, independent of other risk factors [26]. These studies have demonstrated that HR is associated with glucose metabolism.

HR is controlled by the autonomic nervous system (sympathetic and parasympathetic nervous systems). In humans at rest, the parasympathetic tone predominates, and therefore, resting HR is lower than the intrinsic rate of the sinoatrial node [27]. An increased HR can be due to imbalances in the autonomic nervous system with increased sympathetic activity or reduced vagal tone. The autonomic nervous system is also involved in glucose homeostasis through the modulation of the release of insulin and glucagon. The brain, particularly the hypothalamus and brain stem, modulates pancreatic insulin and glucagon secretion through parasympathetic and sympathetic efferent nerves that innervate pancreatic alpha and beta cells [28]. The sympathetic nervous system plays a predominant role in stimulating the glucagon secretion that counteracts the actions of insulin by stimulating hepatic glucose production and thereby increasing blood glucose levels [29]. The activation of the parasympathetic nervous system lowers glucose levels by stimulating the secretion of insulin from beta cells and suppressing hepatic glucose production [30]. According to an animal study, parasympathetic dysfunction is associated with insulin resistance in the development of early metabolic and cardiovascular alterations [31]. Analysis of data from the Netherlands Study of Depression and Anxiety revealed that increased sympathetic and decreased parasympathetic nervous system activities are associated with metabolic syndrome [32]. Therefore, an autonomic imbalance may be related to both increased resting HR and unfavorable glycemic control.

According to the data from the Diabetes Control and Complications Trial/Epidemiology of Diabetes Interventions and Complications study, a higher HR predicted cardiovascular disease and major adverse cardiovascular events, independent of other risk factors in patients with type 1 diabetes mellitus [33]. A low HR also reduced the risk of patients with type 2 diabetes mellitus developing microalbuminuria, independent of blood pressure [34]. In our previous study, resting HR was also associated with long-term survival in patients with AIS and diabetes mellitus [14]. Rigorous resting HR control in elderly patients with coronary heart disease and diabetes mellitus proved beneficial for both blood glucose control and secondary prevention of coronary heart disease [35]. Further investigations into methods of controlling resting HR as a potential measure to achieve favorable glycemic control and improve cardiovascular outcomes in patients with AIS and diabetes mellitus should be considered.

Because of the propensity of beta blockers to cause insulin resistance and impair glycemic control in patients with diabetes mellitus, the influence of beta blockers in glycemic control remains controversial [36–40]. In the current study, the use of beta blockers in patients with AIS and diabetes mellitus failed to achieve better glycemic control than the nonuse of beta blockers after adjustment for potential confounders (Table 3). In addition to beta blockers, several drugs demonstrated the potential to slow the HR and improve glycemic control. Galantamine is an acetylcholinesterase inhibitor that can prevent the hydrolysis of released acetylcholine and increase the overall amount of acetylcholine. Acetylcholine has the ability to slow the resting HR and stimulate insulin secretion [41]. Low-dose galantamine also alleviates inflammation and insulin resistance in patients with metabolic syndrome [42]. Therefore, it has been deemed a potential antidiabetic agent and an add-on therapy to other oral antidiabetics [43]. Some sodium-glucose cotransporter 2 inhibitors, antidiabetic medications that inhibit the absorption of glucose from the proximal tubule of the kidney, have also been shown to promote parasympathetic nervous activity, thereby decreasing blood pressure and HR [44]. Therefore, in addition to improving glycemic control, these HR slowing agents may also have the potential to improve cardiovascular outcomes for patients with diabetes mellitus and tachycardia.

Many factors are associated with unfavorable glycemic control [45–47]. In the current study, female sex, current smoker, and total cholesterol levels generated an increased risk of unfavorable glycemic control (Table 3), which is consistent with previous studies [48–50]. The risk of malnutrition is highly prevalent among patients who have had a stroke. Stroke severity and dysphagia are factors related to malnutrition [51–53]. Therefore, in this study, because of the tendency of restricted food intake, patients with severe stroke (eNIHSS score > 14) were less likely to have unfavorable glycemic control than those with mild stroke severity (Table 3). Long-term glycemic variability, as determined by variability in HbA1c levels, is associated with an increased risk of cardiovascular disease and microvascular complications [54–56]. In this study, increased mean initial in-hospital HR was also associated with higher glycemic variability, as represented by the standard deviation values of HbA1c levels after discharge (Table 2).

Because of the observational nature of the study, this study has several limitations. The association between mean initial in-hospital HR and glycemic control may not imply a cause–effect relationship. Because the vital signs were collected for the first 3 days of hospitalization, patients who were hospitalized for less than 3 days were excluded from the study; therefore, some patients with mild stroke may not have been included. Additionally, because this study was conducted on a population of patients with AIS and diabetes mellitus, our results may not be applicable to patients with other cardiovascular disease with diabetes mellitus.

## Conclusions

This study addressed the prognostic implications of the initial in-hospital HR on glycemic control. Treatment for augmenting parasympathetic activity and reducing HR, such as with acetylcholinesterase inhibitors, may potentially improve glycemic control and cardiovascular outcomes in patients with AIS and type 2 diabetes mellitus. Further prospective clinical trials should be conducted to determine whether interventions that aim to decrease HR are beneficial for this population.

## Data Availability

The data supporting the findings of the article is available in the Chang Gung Research Databank at Chang Gung Memorial Hospital, Chiayi Branch. These data can be available from the corresponding author (J.D. Lee) after obtaining approval from our local IRB.

## Abbreviations

HbA1c: glycated hemoglobin
HR: heart rate
AIS: acute ischemic stroke
SSI: stroke severity index
NIHSS: National Institutes of Health Stroke Scale
eNIHSS: estimated National Institutes of Health Stroke Scale
eGFR: estimated glomerular filtration rate
bpm: beats per minute
OR: odds ratio
CI: confidence interval
